# Potential and Mechanism of Nobiletin in Diabetes Mellitus and Associated Complications

**DOI:** 10.3390/ph18101528

**Published:** 2025-10-11

**Authors:** Chuyun Zhao, Wenjie Lai, Yu Li, Kinfong Hong, Youhua Xu

**Affiliations:** 1 School of Pharmacy, Faculty of Medicine & State Key Laboratory of Mechanism and Quality of Chinese Medicine, Macau University of Science and Technology, Macau 999078, China; a5m520@163.com (C.Z.); 2230028308@student.must.edu.mo (W.L.); 2 Faculty of Chinese Medicine, State Key Laboratory of Mechanism and Quality of Chinese Medicine, Medical Sciences Division, Macau University of Science and Technology, Taipa, Macau 999078, China; yuli@must.edu.mo (Y.L.); kfhong@must.edu.mo (K.H.); 3 Zhuhai MUST Science and Technology Research Institute, Macau University of Science and Technology, Hengqin, Zhuhai 519031, China

**Keywords:** tangerine peel, nobiletin, diabetes mellitus, diabetic complications

## Abstract

The incidence and mortality of diabetes have increased dramatically in recent decades. New strategies to treat diabetes and its complications with minimal side effects are urgently needed. New monomeric molecules extracted from herbs are an alternative medicine that is being explored as candidate drugs for the treatment of diabetes and its complications. Nobiletin, a citrus flavonoid, has recently received increasing attention in scientific research due to its properties in combating diabetes and its complications, while existing research is scattered and unsystematic. This article summarizes recent studies and reviews the potential therapeutic role of nobiletin in preventing and alleviating diabetes and its complications, aiming to provide promising strategies for the clinical management of diabetes and its complications.

## 1. Introduction

Diabetes mellitus is a prevalent endocrine metabolic disorder worldwide. Recent epidemiological projections estimate that by 2045, the global adult diabetic population will reach approximately 783 million, with 164 million cases anticipated in China alone by 2030 [1,2,3]. Chronic diabetes can lead to a variety of complications. These include microvascular diseases, such as diabetic encephalopathy (DE), diabetic retinopathy (DR), and diabetic nephropathy (DN), as well as macrovascular disease, including cardiovascular disease (CVD) [4]. Approximately 1.5 million people die from diabetes and diabetic complications every year [5]. Diabetes and its complications impose a substantial and escalating economic burden [6,7].

Bioactive phytochemicals, particularly polyphenols, have garnered attention for their broad-spectrum efficacy and minimal toxicity in mitigating risk factors associated with various health disorders. Citrus polymethoxyflavone (PMF), such as hesperidin, naringenin and nobiletin, exert preventive effects on diabetes and its complications via diverse mechanisms [8]. Research has shown that PMFs mitigated obesity in high-fat diet (HFD)-fed rats by regulating insulin homeostasis, serum factors, and gut microbiota diversity, while upregulating key metabolic proteins in brown adipose tissue [9]. PMF-rich citrus extracts, including nobiletin and tangeretin, have been reported to correct gut dysbiosis by reducing the Firmicutes/Bacteroidetes ratio and regulating branched-chain amino acid metabolism, thereby mitigating metabolic syndrome [10]. PMFs mixtures containing nobiletin improve glucose tolerance and reduce insulin levels in hypercholesterolemic, hyperinsulinemic hamsters [11]. Among the major PMFs, hesperetin has been reviewed as a promising treatment option for diabetes and related complications [12,13], and numerous reviews have explored the application of naringenin in diabetes and other related metabolic diseases [12,14]. Nobiletin possesses diverse pharmacological properties. In addition to its anti-tumor, anti-inflammatory, immunomodulatory, against respiratory diseases, and neuroprotective effects [15,16,17], numerous in vivo and in vitro studies have also highlighted its potent anti-diabetic potential. However, the pharmacological properties of nobiletin and the molecular basis of its effects on diabetes and its complications have not yet been explored, and further research is still needed. This review summarizes recent research advances regarding the role of nobiletin in the treatment of diabetes and its complications, provides an overview of the development of related novel delivery systems, aiming to offer a theoretical basis and reference for the development of functional foods enriched with nobiletin.

## 2. Basic Information on Nobiletin

### 2.1. Chemical Property of Nobiletin

Nobiletin is a polymethoxyflavone predominantly found in citrus fruits including sweet orange (*Citrus sinensis*), mandarin (*Citrus reticulata*), and lemon (*Citrus limon*). Chemically identified as 5,6,7,8,3′,4′-hexamethoxyflavone (C_21_H_22_O_8_, molecular weight 402.39 g/mol), it possesses a C_6_-C_3_-C_6_ flavone core with six methoxy substituents, conferring high lipophilicity and distinctive bioactivity. Nobiletin exhibits characteristic absorption in the ultraviolet-visible (UV-Vis) region, with its maximum absorption peak typically observed around 330 nm [18]. Owing to its favorable bioavailability and broad pharmacological profile, nobiletin holds considerable commercial and therapeutic value. Due to its elevated methoxy content, nobiletin exhibits pronounced lipophilicity and hydrophobicity, enabling facile solubility in organic solvents such as ethanol and dimethyl sulfoxide. However, it demonstrates poor aqueous solubility (less than 1 μg/mL) and low oral bioavailability (approximately 1%) [19,20,21,22]. Following administration, nobiletin progressively accumulates in the gastrointestinal tract, blood plasma, small intestine, liver, kidneys, and urine. Pharmacokinetic evaluations indicate superior plasma exposure metrics compared to other citrus peel constituents, highlighting its systemic bioavailability [23]. Notably, elevated concentrations in the gastrointestinal muscular layer and liver suggest absorption primarily occurs via the gastrointestinal basement membrane, facilitating entry into systemic circulation and subsequent hepatic accumulation [24]. The absence of glycosidic moieties enhances intestinal absorption efficiency [25,26]. Caco-2 cells, which exhibit morphological and functional similarities to human small intestinal epithelial cells, have been extensively utilized to assess drug absorption potential [27]. Nobiletin displays high permeability in Caco-2 models and is absorbed through proton-linked carboxylate transporters [28]. More importantly, nobiletin has a good safety profile and does not cause any long-term toxicity or significant reduction in body and liver weight [29,30,31,32]. Oral administration of 0.05% nobiletin over 3–20 weeks induced no substantial changes in body weight, liver mass, splenic morphology, or behavioral parameters, indicating minimal adverse effects [33].

### 2.2. Bioactivity of Nobiletin

Nobiletin exhibits protective effects across multiple pathological conditions, including type 2 diabetes mellitus (T2DM) [34], osteoporosis-related bone loss [29], various cancers [19] and hypercholesterolemia [35]. It also confers neuroprotection by attenuating neuronal degeneration and cognitive deficits [36]. Its therapeutic scope extends to components of metabolic syndrome such as cardiovascular disease, visceral obesity, and hypertension. As a bioactive constituent of traditional Chinese medicines like *Citrus aurantium* and *Citrus reticulatae*, nobiletin exerts anti-inflammatory, antioxidant, lipid-regulatory, and insulin-sensitizing effects [37].

Pharmacodynamically, nobiletin inhibits NF-κB activation and attenuates lipopolysaccharide (LPS)-induced inflammation, thereby protecting against acute lung injury [38]. Notably, immature citrus peels contain higher nobiletin concentrations than mature fruit, enhancing its industrial relevance [39,40]. Collectively, these properties position nobiletin as a promising candidate for prevention and management of metabolic syndrome and associated disorders.

## 3. Therapeutic Potential and Mechanism of Nobiletin on Diabetes and Its Complications

**Figure 1:**
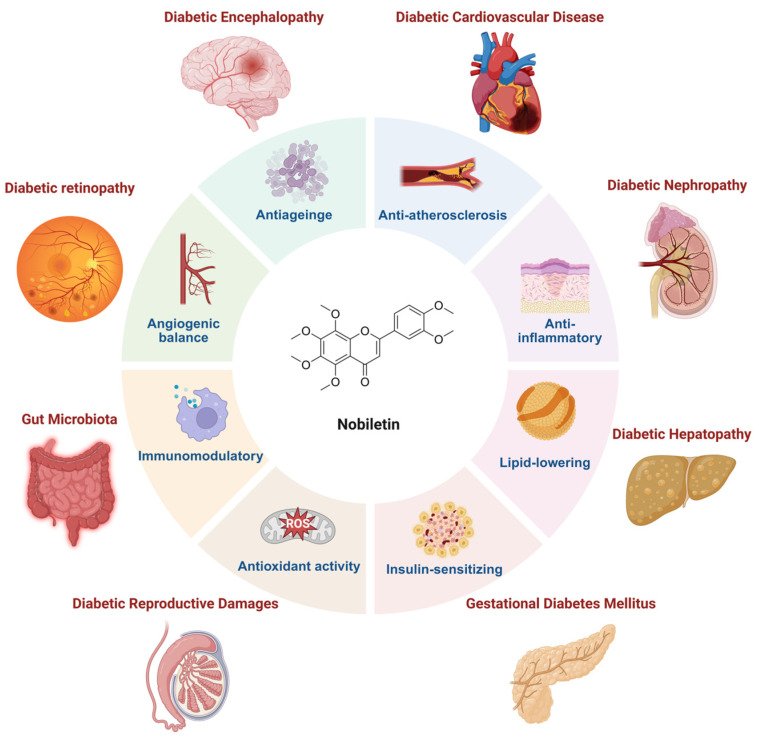
Application of nobiletin in diabetes and its complications. (created with BioRender.com).

Nobiletin has emerged as a promising natural compound with significant therapeutic potential for managing type 2 diabetes mellitus and its debilitating complications (Figure 1). Inflammatory response is an important factor in the progression of diabetes and metabolic diseases [41]. Nobiletin exerts potent anti-inflammatory effects in LPS-stimulated RAW264.7 macrophages by enhancing autophagy, thereby reducing pro-inflammatory mediators including inducible nitric oxide synthase (iNOS) and cyclooxygenase-2 (COX-2). This is mediated via activation of the IL-6/STAT3/FOXO3a signaling axis, promoting macrophage autophagy [42].

Hyperglycemia upregulates chronic inflammatory markers and increases the production of ROS, while prolonged oxidative stress and chronic inflammation can lead to the development of diabetes [43,44]. Chronic low-dose nobiletin supplementation prevents HFD-induced inflammation, insulin resistance, dyslipidemia [45]. In hyperglycemic HepG2 cells and mouse primary hepatocytes, nobiletin activated adenylate-activated protein kinase (AMPK) and restored the phosphorylation of acetyl-CoA carboxylase (ACC), thereby attenuating lipogenesis and promoting fatty acid oxidation [46]. Notably, although AMPKβ1-deficient hepatocytes lost ACC phosphorylation, nobiletin maintained its effects on lipogenesis and fatty acid oxidation. Acute injection of nobiletin into mice did not increase hepatic AMPK or ACC phosphorylation, but effectively prevented HFD-induced obesity, hepatic steatosis, dyslipidemia, and insulin resistance, indicating that its metabolic benefits are independent of hepatic or adipocyte AMPK activation [46]. On the other hand, activation of PPARγ improved insulin sensitivity in adipose tissue and muscle, while activation of PPARα promoted fatty acid oxidation and reduced lipotoxicity, thereby reducing the impairment of insulin signaling [47]. Downstream AMPK is mediated, thereby enhancing glucose uptake by skeletal muscle and adipose tissue, thereby promoting GLUT4 translocation [48]. In obese diabetic models (ob/ob mice), nobiletin significantly increased the levels of adiponectin, PPARα, and PPARγ by regulating the expression of glucose transporters (Glut1, Glut4) in adipose and skeletal muscle tissues, as well as the adipokine profile, thereby improving blood glucose control and insulin sensitivity and inhibiting proinflammatory cytokines (TNF-α, IFN-γ, IL-1β, IL-6) [11,49]. In summary, the mechanisms by which nobiletin regulates metabolism appear tissue-specific and context-dependent. Both AMPK-dependent and AMPK-independent pathways may coexist, collectively contributing to its overall benefits in improving insulin resistance and correcting lipid metabolism disorders.

Nobiletin modulates crosstalk between adipocytes and macrophages, suppressing inflammatory mediator secretion and adipogenic transcription factors via induction of heme oxygenase-1 (HO-1), which is essential for its anti-inflammatory action [50]. It mitigates postprandial hyperglycemia by enhancing hepatic glycolysis and glycogen synthesis while suppressing gluconeogenesis through starch interaction [51]. Nobiletin inhibits intestinal fructose absorption [52], protects pancreatic islets by reducing HIF-1α and ROS, and stimulates adipocyte lipolysis via the cAMP/PKA/hormone-sensitive lipase (HSL) pathway [53,54]. In adipocytes, nobiletin augments insulin-stimulated glucose uptake through activation of PI3K, AKT, and PKA pathways [55]. It promotes adiponectin synthesis during adipocyte differentiation, enhancing insulin sensitivity and exerting anti-atherogenic effects [56]. Mechanistically, nobiletin inhibits adipogenesis by decreasing intracellular triglycerides, downregulating PPARγ2 expression, reducing CREB phosphorylation, and increasing STAT5 phosphorylation, collectively impairing adipocyte differentiation [57,58]. Additionally, nobiletin ameliorates obesity-associated muscle atrophy by enhancing muscle fiber size and myogenic protein expression [59].

In addition to its anti-inflammatory effects, nobiletin also has significant metabolic regulatory effects. It modulates AdipoR1/gp91(phox) expression [60], activates LXRα to enhance ABCA1 and ABCG1 expression through the LXRα-PPARγ axis [61], and promotes bile acid synthesis via CYP7A1 upregulation [62]. Nobiletin binds to docosahexaenoic acid (DHA) and synergistically inhibits nitric oxide production in macrophages by suppressing ERK/p38 phosphorylation and NF-κB nuclear translocation, mediated by complementary pathways [63].

In summary, nobiletin has the effects of lowering blood sugar, improving insulin resistance, protecting pancreatic β-cell function, regulating lipid metabolism disorders, anti-inflammatory and antioxidant effects (Table 1).

### 3.1. Nobiletin Attenuates Gut Microbiota Dysbiosis

Gut microbiota dysbiosis is critically implicated in obesity, metabolic disorders, and intestinal dysfunction, representing a key therapeutic target in diabetes spectrum diseases. Nobiletin ameliorates HFD-induced metabolic dysfunction and hepatic pathology by enhancing insulin sensitivity and normalizing intestinal lipogenesis [64]. It upregulates hepatic CYP7A1, modulating bile acid synthesis and composition potentially via microbiota-dependent mechanisms [65].

In hyperglycemic *ApoE*^−/−^ mice, nobiletin administration for 24 weeks reduced fasting glucose and glycated serum protein levels, improved pancreatic function, and restored gut microbiota diversity, influencing lipid and amino acid metabolism as well as secondary bile acid profiles [66]. Nobiletin administration in HFD-fed mice attenuates weight gain, improves glucose tolerance, restores lipid metabolic disorders, and reverses HFD-induced gut dysbiosis [67]. *Citrus depressa* peel extract containing nobiletin decreased HFD-induced obesity partly through AMPKα activation and modulation of gut microbiota, resembling a prebiotic effect [68].

Nobiletin protects intestinal barrier integrity, as demonstrated by enhanced delivery via yeast microcapsules, which ameliorated ulcerative colitis in murine models through NLRP3 inflammasome inhibition and macrophage polarization modulation [69]. Dietary nobiletin restored antibiotic-induced gut dysbiosis, improved epithelial pathology, increased tight junction protein expression, reduced serum LPS and pro-inflammatory cytokines, and restored short-chain fatty acid and bile acid metabolism [70,71]. In streptozotocin-challenged mice, nobiletin suppressed hyperglycemia via gut microbiota regulation, mitophagy activation, inflammasome inhibition, and islet preservation [72].

### 3.2. Nobiletin Modulates Metabolic Rhythms

Nobiletin modulates circadian rhythms, contributing to its therapeutic efficacy in metabolic and neurological disorders. It enhances circadian amplitude and phase-resetting of core clock genes in hepatocytes, attenuating palmitate-induced lipogenesis via IRS-1/AKT signaling and AMPK-Sirt1 pathways in a *Bmal1*-dependent manner. Nobiletin also ameliorates mitochondrial dysfunction by reducing ROS and restoring respiratory complex expression [73]. In diet-induced obese mice, nobiletin mitigates metabolic syndrome and increases energy expenditure in a Clock gene-dependent fashion; similar clock-dependence is observed in *db/db* mice [74]. It lengthens PER2::LUC oscillation period and amplitude [75]. In pancreatic islets from T2DM donors, nobiletin rescues dampened circadian amplitude and enhances insulin secretion, requiring *Bmal1* expression [76,77]. In adipocytes, nobiletin activates ROR nuclear receptors, enhancing circadian oscillations of clock genes and suppressing lipid accumulation via circadian regulation of adipogenic genes, an effect abolished by RORα/γ knockdown [78]. Hepatic *Bmal1* deletion studies reveal that the triglyceride-lowering effect of nobiletin is independent of *Bmal1*, whereas cholesterol regulation depends on hepatic *Bmal1* [79]. Moreover, nobiletin suppresses adipogenesis through clock-amplified Wnt signaling. It augmented circadian amplitude in preadipocytes, inhibited adipogenic commitment/differentiation via Wnt pathway reactivation, and reduced adipocyte hypertrophy in vivo-effects abolished in clock-disrupted models [80]. Neurologically, nobiletin attenuates neurodegeneration in a Parkinson’s disease rat model by enhancing *Bmal1* and activating the *Bmal1*/NRF2 axis, reducing ferroptosis and oxidative stress, and promoting mitophagy/autophagy [81]. In zebrafish depression models, nobiletin alleviates depressive-like behavior by activating glycolytic signaling via pyruvate kinase M2 [82].

### 3.3. Nobiletin Ameliorates Insulin Resistance

Nobiletin significantly ameliorates insulin resistance (IR), a central pathogenic factor in T2DM. In high-calorie diet-fed mice, nobiletin lowers blood glucose, insulin, and leptin, while reducing adipocyte size [83].

**Table 1:** Summary of Nobiletin for the Treatment of Diabetes Mellitus.

Type of Study	Study Subject	Dose/Dosing Method/Period	Effect and Molecular Mechanisms	References
In vivo	HFD-induced C57BL/6J mice	Nobiletin; 10 or 100 mg/kg; 5 weeks; p.o.	NOB altered the expression levels of several lipid metabolism-related and adipokine genes, increased the mRNA expression of *Pparg*, sterol regulatory element-binding protein-1c, fatty acid synthase, stearoyl-CoA desaturase-1, *Ppara*, *Cpt1a*, *Ucp2* and adiponectin, and decreased the mRNA expression of *Tnf-α* and monocyte chemoattractant protein-1 in WAT. NOB also up-regulated GLUT4 protein expression and AKT phosphorylation and suppressed IκBα degradation in WAT.	[34]
In vitro	LPS; RAW264.7 cells	Nobiletin; 10 μM, 25 μM and 50 μM	Nobiletin also inhibits LPS-induced COX-2, iNOS, and IL-6 expression, suppresses NO production, inhibits phosphorylated STAT3 protein expression, and upregulates p-FOXO3a expression.	[42]
In vitro	3T3-F442 preadipocytes	Nobiletin; 5, 20 or 50 μM	Nobiletin treatment significantly increased the uptake of [(3)H]-deoxyglucose by differentiated 3T3-F442A adipocytes, and this increase is concentration-dependent and related to the PI3K/AKT/PKA pathway.	[55]
In vivo	ob/ob mice	Nobiletin; 200 mg/kg; 5 weeks; p.o.	Nobiletin significantly reduced the mRNA expression levels of inflammatory adipokines such as *Il-6* and *Mcp-1* and increased the mRNA expression levels of adiponectin and *Pparg*, and increased glucose utilization in WAT and muscle.	[49]
In vitro	Insulin, 3-isobutyl-1-methylxanthine (IBMX), and dexamethasone; 3T3-L1 cells	Nobiletin; 16, 32 or 64 μM [57]; 1, 10 or 100 μM [58]; 10 μM [78]	Nobiletin increases the secretion of the insulin-sensitizing factor adiponectin and reduces the secretion of the insulin-resistant factor MCP-1 in 3T3-L1 adipocytes. Nobiletin significantly suppressed the differentiation of 3T3-L1 preadipocytes into adipocytes, upon induction with insulin together with a cAMP elevator such as IBMX, by downregulating the expression of the gene encoding *Pparg2* [57]. In addition, nobiletin decreased the phosphorylation of CREB and strongly enhanced the phosphorylation of STAT5 [58]. Nobiletin induced lipolysis in adipocytes by the activation of the cAMP/PKA/HSL pathway [54]. NOB enhanced the oscillation of core clock genes (*Bmal1*, *Cry1*, *Dec1*, and *Dec2*) in differentiated 3T3-L1 adipocytes, inhibited lipid accumulation in 3T3-L1 and SVF cells, upregulated the expression of IκBα, a target of RORs to inhibit NF-κB activation and proinflammatory cytokine expression [78].	[54,57,58,78]
In vivo	High-fat diet; *Ampkβ1*^−/−^, *AccDKI*, *iβ1β2AKO mice*	Nobiletin; 0.3%, *w*/*w*; 12 or 18 weeks; p.o.	Nobiletin increased phosphorylation of AMPK and ACC in primary mouse hepatocytes, which is associated with increased FA oxidation and attenuated FA synthesis. Despite loss of ACC phosphorylation in *Ampkβ1*^−/−^ hepatocytes, nobiletin suppressed FA synthesis and enhanced FA oxidation. In mice fed a high-fat diet, nobiletin robustly prevented obesity, hepatic steatosis, dyslipidemia, and insulin resistance, and it improved energy expenditure in *Ampkβ1*^−/−^, *AccDKI*, and *iβ1β2AKO* mice to the same extent as in WT controls.	[46]
In vivo	High-fat diet; C57BL/6J	Nobiletin; 0.02%, *w*/*w*; 16 weeks; p.o.	NOB significantly reduced hepatic lipid droplets and triglyceride levels and the expression of inflammatory factors (IFN-γ, TNF-α, IL-6, IL-1β, NF-κB, TLR2, and TLR4). It also improved glucose tolerance and insulin resistance, and decreased plasma insulin, free fatty acids, TC, non-HDL-C, and apolipoprotein B levels.	[45]
In vivo	High-fat diet; *Ldlr*^−/−^ mice	Nobiletin; 0.3%, *w*/*w*; 10 or 12 weeks; p.o. [64]. 0.1 or 0.3%, *w*/*w*; 8 or 26 weeks; p.o. [83]	Nobiletin reduced fasting jejunal triglyceride accumulation through accelerated TRL secretion and lower jejunal fatty acid synthesis with no impact on fatty acid oxidation. Nobiletin led to higher levels of p-AKT and FoxO1 and normal Srebf1c expression indicating increased insulin sensitivity [64]. Nobiletin attenuated dyslipidemia through a reduction in VLDL-triglyceride (TG) secretion. Nobiletin prevented hepatic TG accumulation, increased expression of *Pgc1α* and *Cpt1α*, and enhanced fatty acid β-oxidation. Nobiletin increased hepatic and peripheral insulin sensitivity and glucose tolerance and dramatically attenuated atherosclerosis in the aortic sinus [83].	[64,83]
In vivo	High-fat diet; *ApoE*^−/−^ mice	Nobiletin; 50, 100, and 200 mg/kg; p.o.	The levels of FBG and GSP in hyperglycemic mice are effectively reduced. The secretory function of pancreas is improved. Meanwhile, Nobiletin treatment restored the gut microbial composition and affected metabolic function.	[66]
In vitro	Human pancreatic islet cells	Nobiletin; 20 μM [76] and 10 μM [77]	Nobiletin, an agonist of the core-clock proteins RORα/γ, boosted both circadian amplitude of T2DM islet clocks and insulin secretion by these islets [76]. Nobiletin increased islet circadian clock amplitude and augmented glucose-stimulated insulin secretion (GSIS) in isolated human islets in a *Bmal1*-dependent manner [77].	[76,77]
In vivo	*Bmal1^flox/flox^* and *Bmal1L* KO mice	Nobiletin; 200 mg/kg; p.o.	Nobiletin inhibited hepatic DNL and decreased liver TG in HFD-fed mice independently of liver *Bmal1*, whereas liver-specific Bmal1 depletion reversed the beneficial effects of Nobiletin on liver cholesterol homeostasis, increased serum VLDL levels in *Bmal1L* KO mice, lower liver and serum TC and TG in *Bmal1^flox/flox^* mice.	[79]

Nobiletin also exerts direct hypoglycemic effects. In STZ-induced diabetic mice, it suppresses hyperglycemia by restoring gut microbiota balance, activating mitophagy, inhibiting inflammasomes, and protecting pancreatic islets [72]. Its anti-inflammatory properties counteract IR drivers by suppressing pro-inflammatory cytokines and signaling pathways (NF-κB, JNK/MAPK) and reducing ROS in muscle cells exposed to palmitate [84].

#### 3.3.1. Preservation of Islet β-Cells and Release of Insulin from β-Cells

Nobiletin protects pancreatic β-cells, which are intrinsically susceptible to oxidative stress due to low antioxidant enzyme expression [85]. In vivo, Shiikuwasha extract with high nobiletin content improves glucose tolerance, reduces serum glycoalbumin, and increases β-cell area [86]. In INS-1 β-cells, nobiletin enhances glucose-stimulated insulin secretion via Epac-dependent pathways and inhibits ER stress-induced apoptosis through PKA-mediated suppression of caspase-3 and JNK activation [87]. Mechanistically, β-cell-specific *Bmal1* overexpression enhanced the effects of nobiletin on pancreatic circadian rhythms and insulin secretion, highlighting the key role of *Bmal1* in interventions in diabetes [77]. NIT-1 β-cells studies further confirmed that nobiletin restores mitochondrial membrane potential and inhibits inflammatory pathways [72].

#### 3.3.2. Suppression of α-Glucosidase to Reduce Intestinal Glucose Absorption

Nobiletin, a major flavonoid from a byproduct of the Clementine citrus fruit, exhibits multi-target enzyme inhibitory activity relevant to diabetes management. In vitro studies have shown that nobiletin (50–500 µg/mL) significantly inhibits ROS production in CCD-18Co cells under both physiological and oxidative stress conditions, including inhibition of α-glucosidase and α-amylase [88].

### 3.4. Nobiletin and Diabetic Complications

**Figure 2:**
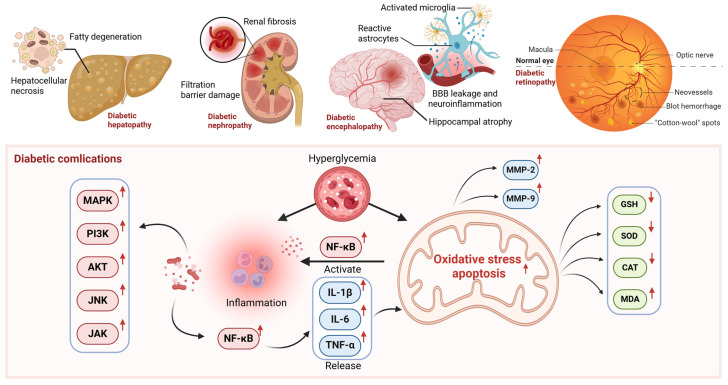
The role of oxidative stress and inflammation in diabetic complications. (created with BioRender.com). Abbreviations: Akt, Protein Kinase B; BBB, Blood–brain Barrier; CAT, Catalase; GSH, glutathione; IL-1β, Interleukin-1 beta; IL-6, Interleukin-6; JAK, Janus Kinase; JNK, c-Jun N-terminal Kinase; MDA, Malondialdehyde; MMP-2, Matrix Metalloproteinase-2; MMP-9, Matrix Metalloproteinase-9; NF-κB, Nuclear Factor Kappa-light-chain-enhancer of activated B cells; PI3K, Phosphoinositide 3-Kinase; SOD, Superoxide Dismutase; TNF-α, Tumor Necrosis Factor-α.

**Figure 3:**
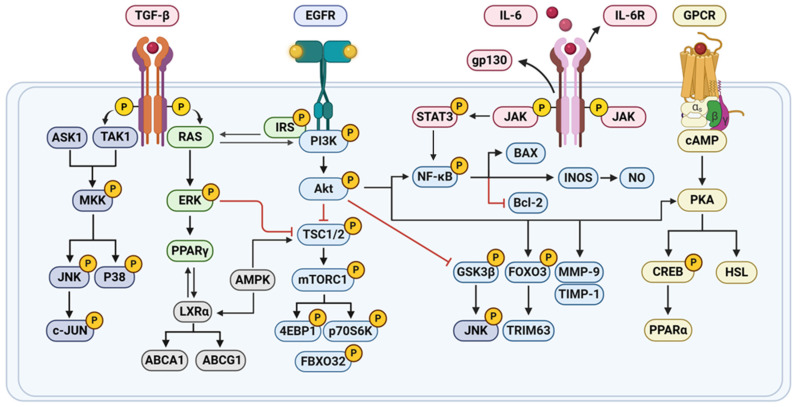
Mechanism of nobiletin in treating diabetes. (created with BioRender.com). Abbreviations: 4EBP1, Eukaryotic Translation Initiation Factor 4E-Binding Protein 1; ABCA1, ATP-binding Cassette Sub-family A Member 1; ABCG1, ATP-binding Cassette Sub-family G Member 1; AMPK, AMP-activated Protein Kinase; ASK1, Apoptosis Signal-regulating Kinase 1; BAX, BCL2-Associated X Protein; Bcl-2, B-cell Lymphoma 2; C-JUN, Jun Proto-Oncogene; cAMP, Cyclic Adenosine Monophosphate; CREB, cAMP Response Element-Binding Protein; EGFR, Epidermal Growth Factor Receptor; ERK, Extracellular Signal-Regulated Kinase; FBXO32, F-box Protein 32; FOXO3, Forkhead Box O3; GPCR, G Protein-Coupled Receptor; gp130, Glycoprotein 130; GSK3β, Glycogen Synthase Kinase 3 beta; HSL, Hormone-sensitive Lipase; IL-6R, Interleukin-6 Receptor; iNOS, Inducible Nitric Oxide Synthase; IRS, Insulin Receptor Substrate; LXRα, Liver X Receptor alpha; MKK, Mitogen-activated Protein Kinase Kinase; mTORC1, Mechanistic Target of Rapamycin Complex 1; NO, Nitric Oxide; p38, p38 Mitogen-activated Protein Kinase; p70S6K, Ribosomal Protein S6 Kinase beta-1; PKA, Protein Kinase A; PPARα, Peroxisome Proliferator-Activated Receptor alpha; PPARγ, Peroxisome Proliferator-Activated Receptor gamma; RAS, Rat Sarcoma; STAT3, Signal Transducer and Activator of Transcription 3; TAK1, TGF-β-Activated Kinase 1; TGF-β, Transforming Growth Factor-beta; TIMP-1, Tissue Inhibitor of Metalloproteinases-1; TRIM63, Tripartite Motif-containing 63; TSC1/2, Tuberous Sclerosis Complex 1/2.

Prolonged hyperglycemia can lead to damage across multiple organ systems in diabetes patients, increasing the risk of severe complications such as diabetic hepatopathy (DH), diabetic nephropathy (DN), diabetic encephalopathy (DE), and diabetic retinopathy (DR) (Figure 2). Numerous studies have demonstrated that nobiletin combats diabetes and its complications through various mechanisms (Figure 3), including anti-inflammatory, antioxidant, or other effects (Table 2).

#### 3.4.1. Nobiletin and Diabetic Hepatopathy

**Figure 4:**
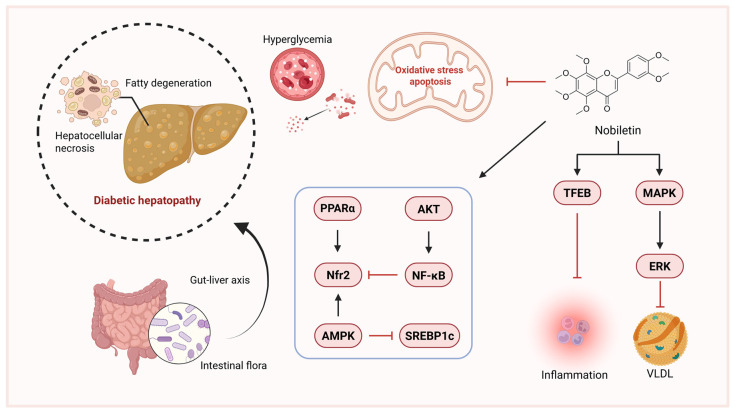
Mechanism of nobiletin in treating diabetic hepatopathy. (created with BioRender.com). Abbreviations: Nfr2, Nuclear Factor Erythroid 2-related Factor 2; VLDL, Very Low-Density Lipoprotein; SREBP1c, Sterol Regulatory Element-binding Protein 1c.

The liver, a central metabolic organ, is often affected in diabetes, notably through NAFLD. NAFLD is bidirectionally linked with diabetes and increases risks of NASH and fibrosis [89,90,91]. Numerous studies corroborate nobiletin’s efficacy in preventing and treating NAFLD [92]. Nobiletin modulates key signaling pathways including Nrf2, NF-κB, AMPK, PPARα, ERK, AKT, and TFEB, contributing to its hepatoprotective effects (Figure 4). It improves liver function, reduces inflammation and oxidative stress, remodels gut microbiota, and mitigates hepatocellular necrosis and insulin resistance, positioning it as a promising therapeutic agent for liver diseases [16]. Nobiletin inhibits apoB100 secretion from HepG2 cells through activation of MAPK/ERK. Dietary nobiletin supplementation attenuates hepatic VLDL overproduction, ameliorates dyslipidemia, and prevents steatosis in HFD-fed *Ldlr*^−/−^ mice by suppressing SREBP1c-mediated lipogenesis and enhancing fatty acid β-oxidation, thereby improving insulin sensitivity and glucose homeostasis [83].

#### 3.4.2. Nobiletin and Diabetic Nephropathy

**Figure 5:**
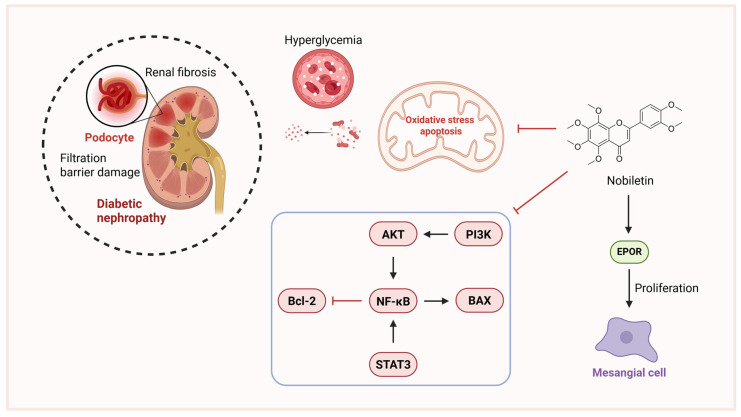
Mechanism of nobiletin in treating diabetic nephropathy. (created with BioRender.com). Abbreviations: EPOR, Erythropoietin Receptor.

Podocytes serve as the final filtration barrier in glomeruli and are critical in diabetic nephropathy (also known as diabetic kidney disease, DKD) pathogenesis [93]. Autophagy in podocytes mitigates oxidative damage in low-glucose environments but is impaired under hyperglycemia [94,95]. Nobiletin attenuates renal injury through multiple mechanisms, including suppression of STAT3/NF-κB signaling, reducing inflammation and extracellular matrix accumulation in hyperglycemic mesangial cells [96]. In STZ/cadmium-induced diabetic nephropathy rats, nobiletin improves glucose metabolism, restores renal function, reduces oxidative stress, and ameliorates histopathological damage by inhibiting Bax expression and NF-κB nuclear translocation, upregulates Bcl-2 to modulating apoptosis regulators [97]. Nobiletin also exhibits anti-fibrotic effects in chronic kidney disease models by inhibiting the galectin-1/PI3K/AKT pathway, reducing renal fibrosis [98]. Additionally, nobiletin functions as a novel erythropoietin receptor (EPOR) agonist, promoting mesangial cell proliferation and anti-apoptotic signaling, with EPOR activation essential for its renoprotective effects [99]. Another study demonstrated that nobiletin alleviated renal function decline and fibrosis accumulation, and inhibited the renin-angiotensin system and exhibited antioxidant effects [100]. Mendelian randomization (MR) analysis revealed associations between JUN, CASP3, HIF-1α, and MMP-9 with DN, while in silico docking studies demonstrated that nobiletin exhibits high binding affinity with these targets [101]. This suggests the potential of nobiletin in the treatment of DN (Figure 5).

#### 3.4.3. Nobiletin and Diabetic Encephalopathy

One of the most deleterious chronic microvascular complications of diabetes mellitus affecting the central nervous system (CNS) is diabetic encephalopathy [102]. Beyond peripheral neuropathy, diabetic neuropathies compromise cerebral neural networks, resulting in memory impairment [103]. DE encompasses neuronal injury mediated by glucotoxicity, wherein chronic hyperglycemia potentiates oxidative stress and neuroinflammation, ultimately culminating in neural cell death [104]. This pathology suggests that structural and functional neuronal damage may be the underlying cause of cognitive deficits, although other mechanisms related to neuronal dysfunction may also contribute to cognitive deficits. These cognitive manifestations coincide with blood–brain barrier (BBB) alterations, hippocampal atrophy, and cellular demise induced by oxidative/nitrosative stress [105].

Nobiletin mitigates LPS-induced neuroinflammation in mice by suppressing microglial activation and pro-inflammatory cytokines through MAPK, PI3K/AKT, and NF-κB pathways, while restoring mitochondrial function and redox balance [106]. Extracts containing nobiletin enhance cerebral glucose uptake and antioxidant enzyme activities, supporting neuroprotection [107]. In different models, the neuroprotective effects of nobiletin are associated with memory and learning [108,109], promotion of neuronal survival and synaptic neurotransmission [110,111], improvement of motor dysfunctions [112], and inhibition of neurodegeneration [113]. Neuroinflammation is a central contributor to neurodegenerative disorders such as Parkinson’s and Alzheimer’s diseases [114]. Although the specific role of nobiletin in DE remains underexplored, its established efficacy in mitigating neural injury underscores significant therapeutic potential. This mechanistic foundation affords valuable insights for future investigations targeting DE pathogenesis.

#### 3.4.4. Nobiletin and Diabetic Retinopathy

Diabetic retinopathy, a prevalent microvascular complication, results from chronic hyperglycemia-induced retinal capillary damage, leading to vascular permeability, ischemia, and neovascularization [115,116]. Gelatinases (MMP-2 and MMP-9) serve as potent mediators of pro-inflammatory [117], pro-angiogenic [118,119] and pro-apoptotic signaling cascades [120]. Müller glial cells constitute a principal cellular source of these matrix metalloproteinases in vitreous and retinal tissues during proliferative retinal disorders [121]. Nobiletin attenuates MMP-9 enzymatic activity through dual mechanisms: transcriptional suppression of MMP-9 gene expression and enhanced tissue inhibitor of metalloproteinase-1 (TIMP-1) production in retinal Müller cells. This regulatory action correlates with inhibition of PI3K/AKT signaling pathway. Furthermore, 4′-demethylnobiletin (nobiletin’s primary bioactive metabolite) potentiates the inhibition of MMP-9 enzymatic activity [122]. Additionally, nobiletin alleviates ER stress-induced Müller cell apoptosis and restores angiogenic balance by increasing PEDF expression, preserving retinal function in diabetic conditions [123]. These pharmacological properties position nobiletin as a promising lead compound for novel therapeutic agents targeting matrix metalloproteinases and for the protection of the blood–retinal barrier (BRB) from breakdown in DR.

#### 3.4.5. Nobiletin and Diabetic Cardiovascular Disease

Diabetic cardiomyopathy (DCM) is characterized by myocardial hypertrophy and fibrosis, driven by oxidative stress, endothelial dysfunction, autonomic imbalance, and insulin resistance [124]. Nobiletin reduces intracellular lipid accumulation in adipocytes [57], decreases pro-inflammatory gene expression in adipose tissue of HFD-fed mice [34], and suppresses aortic lesion development in *Ldlr*^−/−^ mice [83]. In STZ-induced diabetic rodent models, nobiletin lowers arterial pressure, reverses bradycardia, improves vascular reactivity, and attenuates myocardial fibrosis and hypertrophy. It enhances antioxidant enzyme activities (SOD, catalase) and reduces oxidative damage markers (MDA) [125]. Nobiletin also modulates cardiac inflammatory cytokines and fibrosis markers by inhibiting NF-κB signaling, collectively preserving cardiac function [126]. Nobiletin attenuates myocardial ischemia–reperfusion injury in diabetic rats by inhibiting ferroptosis and oxidative stress markers, synergizing with ferroptosis inhibitors [127]. Another study also demonstrated that nobiletin inhibited the AT1R/JAK/STAT pathway to alleviate left ventricular dysfunction and remodeling [100].

#### 3.4.6. Nobiletin and Diabetic Reproductive Damages

Diabetes induces testicular damage via hormonal disruption and oxidative stress. In STZ-diabetic rats, nobiletin administration dose-dependently ameliorates testicular injury, normalizes glycemic and inflammatory markers, restores serum insulin and sex hormones, and improves histopathological features. It reverses diabetes-induced downregulation of testicular LH/FSH receptors and steroidogenic enzymes, suppresses lipid peroxidation, enhances antioxidant defenses, and reduces apoptosis [128,129].

#### 3.4.7. Nobiletin and Gestational Diabetes Mellitus

This IDF Diabetes Atlas 10th edition also shows that hyperglycaemia in pregnancy (HIP) affects approximately one in six pregnancies. Hyperglycaemia in pregnancy encompasses pre-existing diabetes in pregnancy, gestational diabetes mellitus (GDM) and overt diabetes in pregnancy (DIP). Pre-existing diabetes in pregnancy includes known type 1 diabetes, T2DM and rarer forms of diabetes. The WHO and ADA classify pregnant women without pre-existing diabetes but fulfilling non-pregnant diabetes diagnostic criteria as having either “diabetes in pregnancy” [130] or “diabetes complicating pregnancy” [131], respectively.] respectively. Nobiletin can increase insulin sensitivity in human skeletal muscle impaired by inflammation and decrease inflammation-induced pro-inflammatory mediators in human placenta and adipose tissue. These effects may be elicited through the NF-κB, Akt and MAPK pathways. Nobiletin improved fasting glucose levels, and reduced inflammation (Ccl2, Cxcl1, IL-1α, IL-1β and TNF-α) in mice placenta and maternal adipose tissue. These findings indicate nobiletin not only improves maternal hyperglycemia but can also improve inflammation associated with GDM [132].

**Table 2:** Summary of Nobiletin for the Treatment of Diabetic Complications.

Type of Study	Study Subject	Dose/Dosing Method/Period	Diabetic Complications	Effect and Molecular Mechanisms	References
In vitro	LPS; BV-2 cells	Nobiletin; 0, 25, 50, 100 μM	Diabetic encephalopathy	NOB inhibited microglial activation and the production of proinflammatory cytokines COX-2, IL-1β, TNF-α, and iNOS. Inhibition of MAPK, PI3K/AKT, and NF-κB signaling pathways alleviated LPS-induced redox imbalance, mitochondrial membrane potential disturbances, and the suppression of mitochondrial respiration-related protein expression.	[106]
In vitro	High glucose (25 mM); Müller glial cells (MIO-M1)	Nobiletin; 0.25, 4, 64 μM	Diabetic retinopathy	Nobiletin inhibits the PI3K/Akt signaling pathway, suppresses MMP-9 gene expression and enhances TIMP-1 production.	[122]
In vitro	Tm or Tg; MIO-M1 cell	Nobiletin; 64 μM	Diabetic retinopathy	Nobiletin has a protective effect on ER stress-induced Müller cell death and enhances the expression of PEDF in Müller cells, thereby potentially protecting the integrity of the BRB.	[123]
In vivo	STZ; Wistar rat	Nobiletin; 10, 25 mg/kg; 4 weeks; p.o.	Diabetic cardiovascular disease	nobiletin ameliorated the hemodynamic parameters, oxidative stress, collagen level, MMP-2 and MMP-9 levels, and vascular reactivity significantly compared with vehicle treated diabetic group.	[125]
In vivo	STZ; C57BL/6 mice	Nobiletin; 50 mg/kg; 11 weeks; i.g.	Diabetic cardiovascular disease	Nobiletin treatment ameliorated cardiac dysfunction in the DCM group, blunted the mRNA expression of NADPH oxidase isoforms *p67^phox^*, *p22^phox^* and *p91^phox^*, and abated oxidative stress, decreased the *Tgfb1*, *Ctgf*, fibronectin, and collagen Iα expressions and blunted cardiac fibrosis.	[126]
In vivo	High-fat diet, STZ; SD rats	Nobiletin; 5 mg/kg, i.p.	Diabetic cardiovascular disease	Nobiletin reduced ACSL4 and NCOA4 expression and inhibited the effect of Erastin or oe-ACSL4 in increasing ACSL4 expression. Alleviation of myocardial ischemia–reperfusion injury in T2DM rats	[127]
In vitro and In vivo	HepG2 cells; High-fat diet; *Ldlr*^−/−^ mice	Nobiletin; 10 μM; 0.1 or 0.3%, *w*/*w*; 8 or 26 weeks; p.o.	Diabetic hepatopathy	Nobiletin inhibits apoB100 secretion from HepG2 cells through activation of MAPK/ERK. attenuated dyslipidemia through a reduction in VLDL-triglyceride secretion. Nobiletin prevented hepatic TG accumulation, increased expression of *Pgc1α* and *Cpt1α*, and enhanced fatty acid β-oxidation. Nobiletin increased hepatic and peripheral insulin sensitivity and glucose tolerance and dramatically attenuated atherosclerosis in the aortic sinus.	[83]
In vitro	High glucose; glomerular mesangial cell	Nobiletin; 5, 10, 20, 30 μM	Diabetic nephropathy	Nobiletin inhibits IL-1β, IL-6, TNF-α, STAT and NF-κB, reducing ECM accumulation.	[96]
In vivo	STZ/cadmium (Cd)-induced; rats		Diabetic nephropathy	Nobiletin improved pathological damage and reduced renal tubular neutrophil infiltration, inhibited the expression of NF-κB p65 protein, downregulated Bax protein expression, and increased Bcl-2 protein expression.	[97]
In vivo	STZ; albino rats	Nobiletin; 10, 25 mg/kg; 30 days; p.o.	Diabetic reproductive damages	Nobiletin decreased glucose, glycosylated hemoglobin (HbA1c), Homeostatic Model of Insulin Resistance (HOMA-IR), and pro-inflammatory cytokines expression, increased insulin, testosterone, luteinizing hormone (LH), and follicle-stimulating hormone (FSH) expression, improved hyperglycemia, reduced pro-inflammatory cytokines, and augmented insulin, testosterone, LH, FSH and CYP17A1, reduced lipid peroxidation and improved the activity of the antioxidant enzymes and AR in testicular tissues of the diabetic group.	[128]
In vivo	TNF; Human placenta, VAT and skeletal muscle cells; Lepr^db/+^ (*db*/+) mice	Nobiletin; 100, 200 μM; 50 mg/kg; 17 days; p.o.	Gestational diabetes mellitus	Nobiletin increased insulin sensitivity in human skeletal muscle impaired by inflammation and decreased inflammation-induced pro-inflammatory mediators in human placenta and adipose tissue, improved fasting glucose levels, and reduced inflammation in mice placenta and maternal adipose tissue. These effects may be elicited through the NF-κB, Akt and MAPK pathways.	[132]

## 4. Nobiletin in Food Applications

Nobiletin, the principal bioactive compound in Citri Reticulatae Pericarpium (*Chenpi*), exhibits low toxicity and traditional use for digestive and anti-inflammatory benefits [133]. Despite its nutraceutical potential, nobiletin faces significant bioavailability challenges due to poor aqueous solubility, chemical instability, extensive pre-systemic metabolism (via intestinal flora/enzymes), and pronounced first-pass effects [134,135]. Consequently, advanced delivery systems represent a critical research focus. Citrus oil composition influences nobiletin’s solubility and emulsification properties [136]. In vitro, nobiletin modulates gut microbiota by increasing *Bifidobacterium* and acetate production [137]. Hepatic demethylation generates bioactive monodemethylated metabolites, potentially more potent than the parent compound [19,20,138,139]. Agricultural applications demonstrate nobiletin enhancement of in vitro porcine embryo development and developmental gene expression [140]. Nobiletin and hot-melt extrusion holds promise for enhancing both the nutritional value and functional properties of rice starch [141]. Fruits containing nobiletin as safer nutritional alternatives for the treatment of neurological disorders [107].

There have been a lot of studies on preparations to improve the bioavailability of nobiletin. Key delivery advances include nanoscale amorphous solid dispersions, improving CNS delivery [142], and BSA/carboxymethyl inulin-stabilized Pickering emulsions (φ_oil_ = 60%) boosting oral bioavailability [143]. Yeast microcapsules enable targeted colonic delivery (20 mg/kg), mitigating DSS-induced colitis in mice via NLRP3 inflammasome regulation and macrophage polarization [69]. Ultrasonically generated nobiletin nanoparticles (521 nm) significantly enhance pancreatic lipase inhibition (90.3% vs. 68.4%) [144]. Soy protein isolate/κ-carrageenan-stabilized HIPEs improve stability, facilitate clathrin-mediated endocytosis/macropinocytosis, and augment anti-inflammatory effects (reduced NO, IL-6, TNF-α) [145]. Therefore, we will summarize the different preparation processes of Nobiletin (Table 3).

### 4.1. Nanoemulsion Delivery Systems

Notably, nobiletin exhibits relatively high bioavailability even via oil suspensions compared to typical flavonoids. A high-loading nobiletin emulsion (1%) is developed in order to enhance the oral bioavailability of nobiletin [146]. A high-loading (1%) nobiletin nanoemulsion significantly outperformed an oil suspension [147], doubling the plasma AUC of nobiletin and its primary metabolite (4′-desmethylnobiletin, 4′-DMN) over 24 h. Diacylglycerol (DAG)-based structured lipids also show promise for encapsulating hydrophobic bioactives to improve bioaccessibility and bioavailability [148].

Ju et al. [149] developed a docosahexaenoic acid-phosphatidylcholine (DHA-PC) nanoemulsion that enhanced the bioavailability of poorly soluble compounds in vitro and in vivo. Nobiletin -loaded nanoemulsions demonstrated small particle size, stability, and faster digestion/serum entry than oil suspensions. Similarly, in another study, nanoemulsions of nobiletin significantly reduced pro-inflammatory mediators (NO, TNF-α, IL-1β, IL-6) in LPS-stimulated macrophages, indicating enhanced anti-inflammatory efficacy [150]. Sun et al. [151] further optimized hydroxypropyl methylcellulose (HPMC)-stabilized nanoemulsions for nobiletin encapsulation by tailoring oil/water phases. HPMC delayed nobiletin migration, improving stability and bioaccessibility. Adding glycerol monolaurate (GML) and whey protein concentrate (WPC) further enhanced encapsulation efficiency, storage stability, and bioavailability by modifying microstructure.

### 4.2. Self-Nanoemulsifying Drug Delivery Systems (SNEDDS)

Qu et al. [152] co-delivered total glucosides of paeony (TGP) and the P-gp inhibitor nobiletin in a SNEDDS for refractory rheumatoid arthritis (RA). This system fully utilized the inhibitory effect of nobiletin and significantly increased the bioavailability of TGP by 435.04%.

### 4.3. Nanoparticles

Nanoparticles (NPs, 1–100 nm) are versatile carriers in drug delivery. Nanoparticles offer various valuable applications in different fields such as tissue engineering, cell therapy, drug delivery, diagnostic tools, biomaterials, and signaling molecules [153]. Zhang et al. [154] co-encapsulated FTY720 and nobiletin in poly (lactic-co-glycolic acid) (PLGA) NPs for acute lung injury treatment. PLGA NPs enhanced drug solubility, bioavailability, reduced FTY720 toxicity, enabled sustained release, and prolonged lung residence time. Wu et al. [18] synthesized biocompatible, biodegradable metal-phenolic network (MPN) films (~200 nm) for nobiletin delivery in cancer therapy. The MPN stabilized nobiletin, prevented aggregation/crystallization, leveraged the EPR effect for tumor targeting, and enabled controlled release in the tumor microenvironment, improving efficacy and reducing toxicity. Wang et al. [155] utilized MPN to coat nanocrystals, significantly improving nobiletin aqueous stability, preventing crystallization, and enhancing acid stability/release during in vitro digestion. Wu et al. [156] developed zein/tannic acid (TA) NPs for nobiletin. TA addition enabled secondary assembly, stabilizing nobiletin and achieving controlled gastrointestinal release compared to zein NPs alone. Ning et al. [157] formulated a nobiletin solid dispersion (NOB/SD) using Soluplus^®^ and PVP/VA 64 via hot-melt extrusion. NOB/SD markedly enhanced solubility, dissolution rate, bioavailability, cellular uptake, cytoprotection, and antioxidant capacity against acetaminophen-induced liver injury.

### 4.4. Plant-Derived Carriers

In recent years, natural pollen microcapsules have gained increasing attention due to their widespread use as drug delivery vehicles and cell encapsulation scaffolds. Wu et al. [134] exploited sunflower pollen grains (SPG) for nobiletin, achieving an exceptional loading capacity (770 ± 40 mg/g) and demonstrating safety. Wu et al. [158] further engineered SPG microcapsules by loading nobiletin dissolved in medium chain triglyceride (MCT) into SPG and coating with calcium alginate, enabling pH-responsive release (gastric protection followed by rapid intestinal release). Innovatively, green tea infusion (utilizing EGCG and caffeine assemblies via H-bonding/electrostatic interactions) achieves high nobiletin loading (1 mg/mL, >95% encapsulation, >75% loading) and stability, providing a natural food-based delivery platform [159].

### 4.5. Vesicular Systems

Vesicular systems such as liposomes, transfersomes, ethosomes and penetration enhancer-containing vesicles are the most frequently utilized systems for improving topical drug delivery [160]. For topical nobiletin delivery in skin cancer, composite vesicles were developed. Specifically, PEVs combining phospholipids, transcutol as penetration enhancer, and chitosan demonstrated small size, high nobiletin entrapment, and superior skin deposition potential compared to other vesicle types [161].

Nobiletin has been transformed from a traditional medicinal compound into a multifunctional nutraceutical, with its unique methoxyflavone structure supporting its neuroprotective, metabolic regulatory, and anti-aging properties. Advances in delivery technologies and molecular pharmacology are pivotal for clinical translation targeting neurodegenerative diseases, metabolic syndrome, and healthy aging.

**Table 3:** Summary of Nobiletin for Different Delivery Systems.

Delivery Systems	Particle Size	Loading Efficiency/Drug Loading	Encapsulation Efficiency	Release Kinetics	Bioavailability	Reference
Nanoemulsion delivery systems	The averaged droplet size is 325.7 ± 28.1 nm	1% *w*/*v* nobiletin	/	The bioaccessibility of nobiletin delivered by the oil suspension and emulsion is 17.9 ± 1.8% and 81.3 ± 3.0%, respectively.	The bioavailability of nobiletin in oil suspension and emulsion is 19.93 ± 3.93% and 46.20 ± 5.03%, respectively.	[146]
	The average droplet size distribution of the nanoemulsion is 264 nm	The aged citrus peel extract-loaded oil suspension was prepared by suspending 8% of aged citrus extract in pure MCT.	/	The lipids in the nanoemulsion were almost hydrolyzed into free fatty acids at 90 min.	The bioaccessibility of nobiletin in nanoemulsion is 32.3%.	[147]
	The minimum average droplet diameter (about 226 nm)	The nobiletin loading capacity of 1,3-diacylglycerol (1,3-DAG) oil is 14.53 ± 0.44 mg/g.	1,3-DAG oil nanoemulsion showed a high encapsulation efficiency (above 95%).	1,3-DAG releases >80% of free fatty acids (FFA) within 60 min.	The bioaccessibility of nobiletin (above 80%).	[148]
	The average droplet size of the nobiletin emulsion is 196.10 ± 1.16 nm	0.5% *w*/*w* nobiletin	/	The bioaccessibility of naringenin in the oily suspension and emulsion was 13.0 ± 0.2% and 58.6 ± 2.5%, respectively.	The serum Cmax of nobiletin for the emulsion and oil suspension are 1.29 ± 0.03 μg/mL and 0.92 ± 0.04 μg/mL, respectively, and Tmax is 0.5 h.	[149]
Nanoparticles	The size distribution of the nanoparticles is approximately 126 ± 30.18 nm	The drug loading efficiency of nobiletin is 14.93%.	The drug encapsulation efficiency of nobiletin is 74.68%.	The release rate of nobiletin was 10% in 12 h, saturated in 5 days, and 91.4% in 8 days.	/	[154]
	The average particle size of NTFe is 351.2 ± 2.42 nm, and the average particle size of NTAl is 508.6 ± 4.85 nm.	The loading efficiency of NTFe is 286.492%, and that of NTAl is 214.803%.	The encapsulation efficiency of NTFe is 99.914%, and that of NTAl is 99.883%.	The drug release performance of NTFe and NTAl was not much different and was better than that of uncoated nobiletin.	/	[155]
	The particle size of NOB/zein/TA NPs (NZT NPs) is 100 nm.	The nobiletin loading capacity is 48.46 ± 1.62%.	The encapsulation efficiency of NZT48 NPs was 92.37 ± 0.12%.	NZTSZ: Stomach (2 h): Release <10% (resistant to gastric acid degradation). Intestinal (48 h): Slow and sustained release.	/	[156]
	The nobiletin micelles displayed the particle sizes of 68.64 ± 4.37 nm	/	/	NOB/SD releases 95% of nobiletin within 5 min, and maintains supersaturation for at least 120 min.	In the NOB/SD group, Cₘₐₓ is 7.23 ± 1.62 μg/mL and AUC is 9.68 ± 1.74 μg·h/mL.	[157]
Plant-Derived carriers	/	The nobiletin loading capacity of sunflower pollen grains (SPG) is 770 ± 40 mg/g	/	NSGA has a protective sustained release in the stomach (2%) and a long-term sustained release in the intestine (>100 h).	/	[134]
	The particle size of NOB/Tea is around 400 nm	The loading efficiency of nobiletin is over 75%	Encapsulation Efficiency: >95%	/	/	[159]
Vesicular systems	The particle size of composite PEVs is 126.70 ± 11.80 nm	The loading efficiency of nobiletin is 15.08 ± 0.82%.	Entrapment Efficiency: 93.50 ± 3.60%	/	Skin local bioavailability: total skin deposition 95.30 ± 3.40%.	[161]

## 5. Nobiletin and Clinical Trail

Clinical studies corroborate the metabolic benefits of citrus polymethoxylated flavonoids including nobiletin. A 12-week grapefruit intervention reduced insulin levels during glucose tolerance testing in obese individuals [162]. Similarly, a 12-week citrus PMFs extract (900 mg/day) decreased body weight and plasma glucose in overweight subjects [163]. A randomized, double-blind, placebo-controlled trial combining flavonoid-fortified orange juice with caloric restriction in obese participants demonstrated superior reductions in LDL cholesterol, glycated hemoglobin, and systemic inflammation markers, alongside enhanced antioxidant capacity and favorable adipokine modulation (Table 4) [164]. These studies highlight the therapeutic potential of citrus flavonoid mixtures, but further clinical validation is needed to determine the individual effects of nobiletin.

## 6. Nobiletin and Commercial Value

The commercial potential of nobiletin is highlighted by its extensive patent portfolio, with numerous global patents filed to date. These patents cover innovations in extraction, purification, synthesis, and analytical methods, which are crucial for ensuring standardized production and quality control for industrial applications [165,166,167,168,169]. Notably, multiple patents support the therapeutic use of nobiletin in diabetes [170,171] and diabetic cardiomyopathy [172], attributed to its hypoglycemic, hypolipidemic, and anti-inflammatory properties. In the food industry, nobiletin has been patented as a natural preservative [173] and functional food additive [174,175], demonstrating antioxidant, antimicrobial, and flavor-enhancing properties, with demonstrated benefits for metabolic and intestinal health [176,177]. In the cosmetics sector, the patent emphasizes the whitening effect of nobiletin and utilizes its ability to inhibit melanin production [178]. This expanding patent portfolio not only demonstrates nobiletin’s versatility but also signals its high commercial potential. Through continued research into novel delivery technologies and synergistic formulations, nobiletin has the potential to transform from a research-focused phytochemical into a commercially successful ingredient in functional foods, cosmetics, and therapeutic products.

## 7. Conclusions

In conclusion, nobiletin, a prominent polymethoxyflavone abundant in citrus peels, emerges as a multifaceted therapeutic candidate with significant potential for managing diabetes mellitus and its pervasive complications. The escalating global burden of diabetes, projected to affect hundreds of millions globally, underscores the urgent need for innovative treatment strategies. Nobiletin, characterized by its distinctive lipophilic structure and favorable safety profile, addresses core pathophysiological mechanisms across multiple organs affected by diabetes. Its potent anti-inflammatory and antioxidant properties form a foundational basis for its therapeutic efficacy. Crucially, nobiletin combats insulin resistance, protects pancreatic β-cell function, enhances insulin secretion, and modulates key signaling pathways (including AMPK, PI3K/AKT, PPARs, NF-κB, and circadian regulators like *Bmal1*/RORs) to improve glucose and lipid homeostasis.

Beyond glycemic control, nobiletin demonstrates remarkable protective effects against a spectrum of diabetic complications. It attenuates diabetic encephalopathy by mitigating neuroinflammation and oxidative stress, protects against retinopathy by inhibiting MMP-9 and preserving retinal function, alleviates cardiomyopathy by reducing fibrosis and oxidative damage, prevents hepatopathy/NAFLD via metabolic reprogramming, and ameliorates nephropathy by suppressing inflammation and fibrosis. Its unique ability to amplify circadian rhythms further enhances its metabolic benefits. A critical aspect of its action involves modulating gut microbiota composition and function, improving barrier integrity, and influencing bile acid metabolism, thereby addressing gut dysbiosis as a key contributor to metabolic dysfunction. Therefore, it is necessary to focus on the cross-regulatory mechanisms within T2DM signaling pathways and to investigate the effects of nobiletin on systemic metabolic networks, including the gut-liver axis, brain–gut axis, and gut-kidney axis.

While clinical trials support the metabolic benefits of citrus flavonoids, the clinical translation of nobiletin is currently hindered by its inherent poor aqueous solubility and low oral bioavailability. However, significant progress is being made in developing innovative delivery systems (nanoparticles, emulsions, microcapsules) to overcome these limitations and enhance bioaccessibility and targeted delivery. Given its broad-spectrum efficacy, low toxicity, and origin in dietary sources like *Chenpi*, nobiletin is a prime candidate for development into functional foods and nutraceuticals. To ensure successful translation, standardized extraction methods and stringent quality control protocols for nobiletin are essential, along with extensive safety and toxicological evaluations of its novel delivery systems. Future research needs to prioritize elucidating the complete molecular underpinnings of its effects, optimizing delivery strategies, and conducting robust human clinical trials to fully realize the promise of nobiletin as a natural therapeutic agent against diabetes and its complications.

## Figures and Tables

**Table 4:** Summary of nobiletin in the treatment of diabetes in clinical trials.

Treatment	Clinical Trial Program/Identifier	Indication	Development Stage	Clinical Result	Reason for Terminal or Negative Result	Reference
Orange juice (14%: nobiletin, sinensetin, tangeretin)	NCT06680635	Obesity Adult Onset/Diabetes Mellitus Type 2	/	No Results Posted	No results.	NCT06680635
Orange juice (nobiletin, 22.8 mg/L)	NCT06279780	Obesity Adult Onset	/	Consuming a juice containing 22.8 mg/L nobiletin combined with a low-calorie diet significantly improved anthropometric measures and metabolic biomarkers in obese individuals, including lower LDL-C, ApoB/ApoA1 ratio, and A1c levels, enhanced antioxidant capacity and GPX1 expression, accompanied by decreased inflammatory markers such as TNFα and IFNγ, and enhanced adipokines (leptin, PAI-1, and adiponectin).	The short intervention period of 6 weeks; the lack of consideration of other bioactive compounds in the juice; the lack of blood or urine markers to confirm adherence	[164]

## Data Availability

No new data were created or analyzed in this study. Data sharing is not applicable to this article.
